# Should I Pay or Should I Grow? Factors Which Influenced the Preferences of US Consumers for Fruit, Vegetables, Wine and Beer during the COVID-19 Pandemic

**DOI:** 10.3390/foods11111536

**Published:** 2022-05-24

**Authors:** Meike Rombach, David L. Dean, Tim Baird, Jacob Kambuta

**Affiliations:** 1 Department of Land Management and Systems, Lincoln University, Lincoln 7647, New Zealand; jacob.kambuta@lincoln.ac.nz; 2 Department of Agribusiness and Markets, Lincoln University, Lincoln 7647, New Zealand; david.dean@lincoln.ac.nz (D.L.D.); tim.baird@lincoln.ac.nz (T.B.)

**Keywords:** COVID-19, horticultural products, PLS-SEM

## Abstract

This study examines the key factors that determine the preferences of US consumers towards the growing and processing used for horticultural products such as fruit, vegetables, wine and beer over their preferences for buying them both before and after the COVID-19 pandemic. The findings obtained using Partial Least Square Structural Equation Modeling (PLS-SEM) show that engagement with horticulture prior to and after the occurrence of COVID-19 influenced preferences for the growing and processing of fruit, vegetables, wine and beer over buying them in both the pre-COVID-19 and post-COVID-19 contexts. Engagement with horticulture before and after the COVID-19 pandemic was significantly impacted by attitudes towards US growers. Attitudes towards COVID-19 and human values such as self-enhancement, conservation and self-transcendence were also found to be significant factors, while openness to change was not found to be significant. Best practice recommendations are included on the basis of these findings for managers of community gardens, horticultural properties and specialized food stores.

## 1. Introduction

In December 2019, a new variant of the coronavirus known as SARS-CoV-2 caused a global pandemic [1,2]. The highly transmittable virus reached the United States of America (US) during February 2020 and the rapidly growing number in infections resulted in control measures and health mandates being enacted by the US government to counteract the spread of the virus [3,4]. Stay-at-home orders were issued that advised US citizens to avoid socializing and attending mass events. Leaving the house was however permitted for medical appointments and food shopping [5,6,7].

This extended stay-at-home period not only required balancing screen time for work and socialization [8,9,10], but also led to adverse effects on physical and mental health [11]. In order to overcome feelings of unproductiveness and boredom, US citizens discovered new hobbies such as home gardening; this enabled them to take advantage of the space available in their yards, terraces and on their balconies [11,12,13]. Container gardening became one of these new gardening trends; bottles, cans, boxes and other containers were used to grow food. Horticultural blogs and social media profiles of influencers and growers served to provide important sources of information regarding materials, plant varieties and plant production [11]. As a result of this, horticultural retail stores in the US enjoyed a significant increase in sales, particularly in the online shopping environment [14,15]. A large share of these sales was attributed to gardeners buying materials and plants for their home gardens during the pandemic [16].

Despite the growth in gardening as a leisure activity, the production of home-grown food is still very much a critical lifeline for many US citizens in terms of food security and providing regular access to fresh produce [16,17,18,19]. The US is reliant on the global food supply system, which has been disrupted since the occurrence of COVID-19 [3,20,21,22]. Regular chain flows from material suppliers to final consumers were disrupted by the increased cost of freight and the late arrival of cargo delivering imported products [11]. These disruptions caused supply chain uncertainties for consumers, resulting in panic buying and hoarding amongst consumers [5]. This scenario also aided the development of a partial distrust towards global food systems [5]. As both a response and a strategy in terms of preparedness, consumers started growing their own food at home [23,24]. This allowed consumers to sidestep the threat of economic insecurity and to focus on the beneficial effects of their new pursuit of mental well-being. The growth in the number of people who were growing their own produce also served to influence their decision making around whether to buy fresh or processed horticultural products in the future [16].

This study is dedicated to extending the knowledge surrounding these trends through examining US consumer preferences towards growing versus purchasing a variety of horticultural products [25]. In addition to fruit, this study also includes vegetables as fresh products, alongside wine and beer as processed horticultural products, and examines the purchase behavior of US consumers surrounding these products both before and after the occurrence of COVID-19.

This research adds value to the extant literature in this topic area through considering other factors that potentially drive consumer preferences when considering growing over buying horticultural products [25]. This includes a focus on universal human values as defined by Schwartz (1992), given that values determine attitudes and ultimately behaviors. While universal human values have been widely studied as predictors for consumer buying decisions related to food [26,27,28,29,30], the degree to which growing over buying affects these decisions is yet to be explored. Socio-demographic factors were not included in the study, as prior studies indicate that there is no consensus on their significance [31,32,33,34,35]. Recent research investigating the factors that determined the preferences of US consumers towards growing over buying fruit before and after the COVID-19 pandemic showed that socio-demographic factors were largely insignificant [25]. These factors have been excluded from this study as a result of these findings. Suitable predictors, however, included universal human values such as self-transcendence, self-enhancement and participants’ openness to change. These factors were investigated alongside attitudes towards US growers throughout the pandemic and were also examined within the context of the overall engagement that US consumers have with horticulture.

### 1.1. Human Values

A number of consumer studies related to food choices have utilized the theoretical foundations relating to values provided by Schwartz (1992) [26,27,28,29,30]. These studies focus on convenience foods and local foods, whilst also examining organic or sustainable products in terms of the applicability of this theory and the individual values it espouses [36,37,38,39]. Schwartz’s (1992) human values are universally applicable [40,41] and viewed as the criteria that consumers use to justify and evaluate their behaviors and choices [38]. Schwartz (1992) identified ten core values and associated them with a goal that each value stands for. This study also specified the requirements of universality for each value and their relationships to the other core values [40]. The behaviors that resulted from any of these values have consequences that are either in conflict with or conform with another value [41]. These interconnections are presented in a circular structure (see Schwartz (1992) for the definitions of these values) and individual values were presented according to higher order groups [40,41]. Universalism and benevolence were presented as self-transcendence, self-direction, stimulation, hedonism, openness to change, achievement and power as self-enhancement and security tradition and conformity as conservation [40,41]. Within the context of growing one’s own food and being engaged with horticulture, the relationship between higher ordered groups and these individual values is clearly reflected in their motivation to grow food in either community or home gardens [16,42,43,44,45]. Gardeners indicated that ecological concerns, food security, food control and the need for engagement with food production were vital; these four factors were also supported by a desire amongst gardeners to participate in a community and the physical and mental health benefits that resulted from simply being in the garden [11,16].

In a similar manner, these values are also reflected in the reasons why consumers buy organic, sustainable and local food. This is echoed through their food choices which serve as a form of engagement with horticulture [46,47]. While some studies have used these values as predictors of buying behavior, other researchers have used this same set of values to identify consumer segments [38,48]. It has been found that consumers purchasing organic and sustainable food value benevolence, security, hedonism, universalism, self-direction, stimulation and conformity [38]. Other values such as achievement, conservation and universalism are relevant for consumers who strive to make healthier food choices [38]. Another key finding within this area of research is that the most important values predicting pro-social and environmental behavior in the context of gardening are self-transcendence and self-enhancement [49]. However, at times consumers may fail to adhere to their values, which explains the value–behavior discrepancy found in several studies; therefore, values serve as overall orientation principles towards enacted behaviors [49].

### 1.2. Attitudes towards Growers

Consumer attitudes towards horticultural production and horticultural growers in the US are very heterogeneous [50,51]. While some consumers feel that they can trust horticultural growers and have positive attitudes towards them, others simply distrust US growers. These consumers have voiced negative attitudes [52,53], often resulting from a critical perception of production practices or confusion related to concepts such as sustainable or organic production [51]. The two-fold role of horticultural growers, which requires them on the one hand to use land and scarce resources whilst on the other hand to practice stewardship, is often critically regarded by society [15]. Issues in horticultural production such as the treatment of labor and labor conditions, environmental impacts and the use of resources and technology have also led to negative attitudes towards growers [15].

### 1.3. Attitudes towards COVID-19

Since the occurrence of the coronavirus disease, research has focused on the impact that COVID-19 has had on US society, as well as beliefs and attitudes of people towards lockdowns, health mandates, physical restrictions and impact on their everyday life [3,4,5,6,7]. This includes food purchases, gardening and other horticulture-related activities where physical contact and gatherings occur [11,15,16]. Prior studies have found a diverse range of attitudes towards physical distancing, mask wearing and being vaccinated [1,54]. Common factors that are mentioned that affect attitudes include social pressure, rule compliance and free-riding behavior [1,54]. Positive attitudes towards voluntary mask wearing, getting tested and getting vaccinated were found amongst compliant people as these measures benefit public health and also limit the number of hospitalizations and deaths [1]. Negative attitudes were a result of a fear of social shaming, incomplete knowledge about the effectiveness of health measures, controversies over personal rights and freedom, as well as a distrust towards scientists and those in government [54].

### 1.4. Engagement with Horticulture

Extended stay-at-home periods as well as closed borders and travel restrictions resulted in an increased popularity of gardening and other horticultural activities both in the US and globally [11,16,55]. Media and scientific studies alike reported that not only did the sales of seeds, flowers, shrubs and trees increase [16] but also there was an increase in horticultural do-it-yourself projects and bee keeping [11,56]. There was also a dramatic increase in the number of horticulturally themed YouTube videos, social media posts, books, magazines and educational materials all designed to appeal to consumers [11,55]. Tighter budgets resulting from pandemic-related economic hardships alongside the scarce availability of some products resulted in consumers undertaking their own measures to overcome food insecurity, e.g., keeping higher food stocks and home food processing [57,58,59].

### 1.5. Conceptual Model

**Figure 1:**
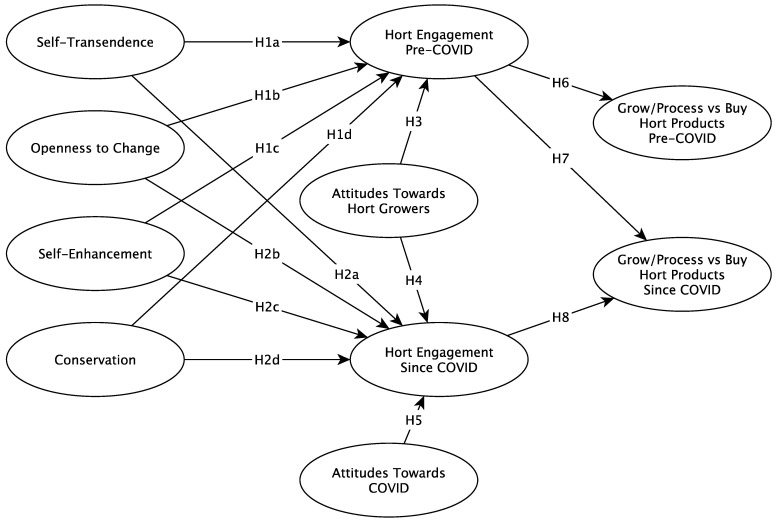
The conceptual model and hypotheses associated with this study.

The conceptual model shown in Figure 1 provides an anchor for the foundations of this study. It shows that US consumers and their preferences regarding the growing and processing of horticultural products such as fruit, vegetables, wine and beer before and after the COVID-19 pandemic is likely to have been influenced by universal human values, attitudes towards horticultural growers and the COVID-19 pandemic and consumer engagement with horticulture. In the pre-COVID-19 scenario, attitudes towards COVID-19 are disregarded. The following eight hypotheses are proposed:

**Hypothesis** **1** **(H1).** *US consumer engagement with horticulture pre-COVID-19 could be impacted by values such as (a) self-transcendence, (b) openness to change, (c) self-enhancement and (d) conservation.*

**Hypothesis** **2** **(H2).** *US consumer engagement with horticulture since COVID-19 could be impacted by values such as (a) self-transcendence, (b) openness to change, (c) self-enhancement and (d) conservation.*

**Hypothesis** **3** **(H3).** *US consumer engagement with horticulture pre-COVID-19 could be impacted by their attitudes towards horticultural growers.*

**Hypothesis** **4** **(H4).** *US consumer engagement with horticulture since COVID-19 could be impacted by their attitudes towards horticultural growers.*

**Hypothesis** **5** **(H5).** *US consumer engagement with horticulture since COVID-19 could be impacted by their attitudes to COVID-19.*

**Hypothesis** **6** **(H6).** *US consumer engagement with horticulture prior to the occurrence of COVID-19 may impact preferences regarding the growing and processing of horticultural products such as fruit, vegetables, wine and beer prior to the COVID-19 pandemic.*

**Hypothesis** **7** **(H7).** *US consumer engagement with horticulture prior to the occurrence of COVID-19 may impact preferences regarding the growing and processing of horticultural products such as fruit, vegetables, wine and beer since the COVID-19 pandemic.*

**Hypothesis** **8** **(H8).** *US consumer engagement with horticulture since the occurrence of COVID-19 may impact preferences regarding the growing and processing of horticultural products such as fruit, vegetables, wine and beer since the beginning of the COVID-19 pandemic.*

## 2. Material and Methods

### 2.1. Survey Instrument

This study is based on data from an omnibus survey consisting of a total of 69 questions covering multiple topics related to the values, beliefs and attitudes of consumers towards horticultural production and the consumption of fruit, vegetables, wine and beer. Data for this study was collected in Fall 2021 using Amazon Mechanical Turk, an online crowd-sourcing platform that is often used within the realm of social sciences [60,61]. The survey was approved by the Human Ethics Committee at Lincoln University (HEC2021-20). Respondents needed to reside in the US and be at least 21 years of age to be able to give informed consent in order to participate in the survey. Further screening questions asked the survey participants to indicate whether they buy fruit and how frequently. For both questions, survey takers who indicated that they did not buy fruit at all were excluded from the study. After data cleaning, a total of 383 surveys were used for the analysis using Partial Least Square Structural Equation Modeling (PLS-SEM). The sample size was deemed appropriate following the ten times rule, which is a commonly used rule in PLS-SEM [62].

Questions used in this study were based on seven attitudinal statements asking survey participants about their attitudes towards horticultural growers and five statements addressing their attitudes towards COVID-19. A seven-point Likert scale ranging from strongly disagree (1) to strongly agree (7) was used. The attitudinal questions focusing on US apple growers featured statements concerning horticultural traditions, grower contributions and the social impact of horticultural production. The focus on horticultural growers is based on the assumption that, from a consumer’s perspective, growers are seen to be in charge of the product and the production processes; they are also viewed as managing the use of land under their stewardship and resources as well [25]. Favorable or unfavorable attitudes are likely to influence the decision whether consumers buy or grow [25]. The questions addressing COVID-19 focused on culture, society, fairness, instability and control, which are common topics in the recent literature within this research domain [1,2,3,15]. Similarly, all nine questions related to horticultural engagement were based on a seven-point Likert scale ranging between strongly disagree (1) and strongly agree (7) and focused on home gardening, bee keeping, food processing and food sharing. Questions were also asked that focused on horticultural education through books, YouTube clips and social media. These questions were influenced by and adapted from Bulgari et al., (2021) [11]. The questions relating to human values were informed by the work of Lindeman and Verkasalo (2005) and respectively covered all ten values using their suggested importance scale [63]. Buying vs. the growing or processing of fruit, vegetables, wine and beer before and after COVID-19 (5 items each) was measured with 0–100 sliding scales ranging from ‘Regularly purchase’ (0) to ‘Grown by myself ‘(100) [25].

### 2.2. Approach and Data Analysis

The data analysis was conducted using the software packages SPSS and SmartPLS. PLS-SEM is known as a suitable approach for exploratory studies where models are highly complex with key driver constructs that need to be identified and where sample sizes are small [64,65]. Three forms of analysis are employed in this approach: path analysis, regression analysis and principal component analysis. Using this approach does not make any distributional assumption on the data concerned [65,66]. Analysis and interpretation of a PLS-SEM model follows a two-stage approach. Reliability and validity are assessed via measurement models (outer model assessment) and the assessment of the structural model (inner model assessment) [67,68,69]. Measurement models represent the relationships between the observed data and the latent variables, whilst the structural model displays whether any relationships exist between the latent variables [65].

For the outer model analysis, construct reliability is considered satisfactory when reliability indicators (Cronbach’s alpha and composite reliability) are greater than 0.6. Convergent validity is reached when items contribute to constructs and these constructs capture item variation. The contribution of items is examined via factor loadings on the appropriate construct. Following Hair (2022), loadings must be greater than 0.4 [65,70]. Likewise, a construct is said to capture sufficient item variation when the average variance extracted (AVE) is greater than 0.6 [65]. The Fornell–Larcker criterion, cross-loadings and the Heterotrait–Monotrait ratio of correlations criterion (HTMT) serve to evaluate discriminant validity. To satisfy the Fornell–Larcker criterion, each construct’s AVE needs to have a square root which is higher than the correlation that it has with another construct and also when the item concerned loads highest on its related construct [71,72]. The HTMT examines the correlations of items within a scale and the correlations between items of different scales, which then enables a ratio to be calculated. If this HTMT ratio is less than 0.9, discriminant validity can be confirmed [73]. The Variance Inflation Factor (VIF) determines whether multicollinearity within the data is an issue and is used when target thresholds are less than 5 [65,66].

Analysis of the inner model, the structural fit, the explanatory power and the model’s predictive relevance are also examined [65]. PLS-SEM convention suggests reporting some Goodness of Fit (GoF) measures, even though Hair et al., (2022) recommend caution when interpreting model fit indices [65]. Measures that summarize model fit such as GoF and Normed Fit Index (NFI) are typically used, with the higher scores indicating the best fit. Standardized Root Mean Square Residual (SRMR) indicates a better fit if these values are small. SRMR values under 0.08 are deemed as acceptable, while those which are over 0.10 are considered unacceptable [65]. Finally, the explanatory power (R^2^) and the predictive validity (Stone–Geisser criterion Q^2^) were checked. R^2^ values are interpreted as weak, moderate or substantial if they are near 0.25, 0.50 and 0.75, respectively. Q^2^ values larger than zero indicate good predictive validity and values higher than 0.25 indicate medium predictive relevance, while those values which are higher than 0.50 indicate strong predictive relevance [65]. Once the inner and outer model analyses have delivered appropriate results, the hypothesis testing can begin.

## 3. Results

Table 1 displays the survey respondents’ socio-demographic backgrounds. The sample consisted of 51.2 per cent men and 48.8 per cent women. The main share of survey respondents resided in the mid-west of the US (34.8 per cent), followed by respondents from the south, northeast and western US at 23.5 per cent, 21.7 per cent and 20.1 per cent, respectively. The median age of respondents was between 25–34 years old. In terms of educational attainment, this group was seen to hold a bachelor’s degree and have an annual pre-tax income of between USD 25,000 and USD 50,000. Although a convenience sample was used, the relevant percentages from the US Census [74,75] were included for reference.

Table 2a–c present the descriptive statistics, factor loadings, reliabilities and convergent validity. Since all factor loadings are greater than 0.4, the constructs each contribute sufficiently to their respective scale. Self-transcendence had a Cronbach’s Alpha of less than 0.6, but all other reliability scores were greater than 0.6, verifying the reliability of the measurement model. With all AVE scores greater than 0.5, convergent validity is also confirmed, satisfying the requirements for construct reliability and convergent validity [65].

Table 3 shows the Fornell–Larcker and HTMT ratios. The cross loadings of all but two of these were less than the square root of the individual constructs’ AVE and similarly, with the exception of two of the HTMT ratios, all were smaller than 0.90. In both cases, the exceptions were the ratio between growing versus buying since COVID-19 and growing versus buying pre-COVID-19 (cross loading: 0.947; HTMT: 1.000), as well as engagement with horticulture since COVID-19 and engagement with horticulture pre COVID-19 (cross loading: 0.870; HTMT: 0.915), which are both higher than recommended. However, this is not an issue, because the two constructs (growing vs. buying and engagement with horticulture) measure the same concept from the two different time perspectives: prior to the occurrence of COVID-19 and since the occurrence of COVID-19. In addition, the largest VIF was 4.105 and the average VIF was 2.142. Multicollinearity was also not an issue with the data as both values are below the recommended threshold of 5 [65]. Therefore, it can be said that, apart from the aforementioned exceptions, discriminant validity is confirmed.

The model can be considered to have an adequate fit due to having a GoF of 0.636, an NFI of 0.680 and an acceptable SRMS of 0.089. The model has moderate explanatory power and strong predictive relevance due to the average R^2^/Q^2^ values of 0.531/0.522. A number of parts of the model were stronger than others, however. The R^2^/Q^2^ scores of 0.567/0.696 for the preference of growing over buying pre-COVID-19 and 0.596/0.693 for the preference of growing over buying since COVID-19 would be considered to show moderate explanatory power and a strong predictive relevance. The values of 0.452/0.623 for engagement with horticulture pre-COVID-19 are considered to be of weak–moderate explanatory power and have a strong predictive relevance, whereas the values of 0.510/0.558 for engagement with horticulture would be considered to have moderate explanatory power and a strong predictive relevance. This confirms that the model is an appropriate fit for hypothesis testing.

**Figure 2:**
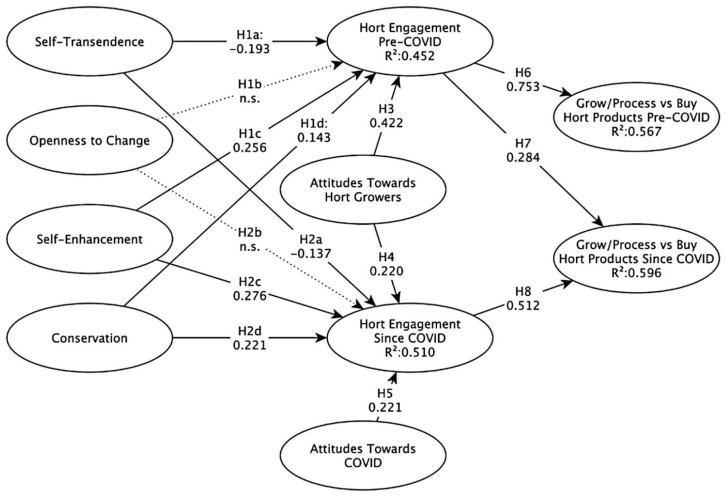
Conceptual Model Results.

The results of the hypothesis testing are shown in Table 4 and Figure 2. Self-transcendence, self-enhancement and conservation were significantly related to engagement with horticulture pre- and post- the occurrence of COVID-19, supporting H1a, H1c, H1d, H2a, H2c and H2d. The value ‘Openness to change’ appears to not be related to engagement in both scenarios and this is indicated by the insignificant relationships for H1b and H2b. Attitudes towards growers and attitudes towards COVID-19 are significantly related concepts to horticultural engagement, supporting H3, H4 and H5. Interestingly, the influence of attitudes towards horticultural growers had a stronger influence on horticultural engagement before COVID-19 than after the pandemic. This could be an indication that attitudes towards COVID-19 are shared with, or are partly replacing, the influence of those horticultural grower attitudes that have emerged since COVID-19.

In addition, engagement with horticulture pre-COVID-19 is related with the preference of growing over buying pre- and post-COVID-19; this is shown by the significant relationship supporting H6 and H7. Similarly, engagement with horticulture since COVID-19 showed a significant relationship with the preference of growing over buying since COVID-19, supporting H8. Additionally, it is interesting to note that horticultural engagement pre-COVID-19 is less influential on the preferences of growing over buying since COVID-19, which further denotes a change in the influence of horticultural engagement since COVID-19.

**Table 4:** Path Coefficients Results.

Hypothesized Relationship	Coefficient	T Stat	*p* Value
H1a: Self-Transcendence -> Hort Engagement Pre-COVID	**−0.193**	3.884	0.000
H1b: Openness to Change -> Hort Engagement Pre-COVID	0.054	0.937	0.349
H1c: Self-Enhancement -> Hort Engagement Pre-COVID	**0.256**	4.214	0.000
H1d: Conservation -> Hort Engagement Pre-COVID	**0.143**	2.434	0.015
H2a: Self-Transcendence -> Hort Engagement Since COVID	**−0.137**	2.833	0.005
H2b: Openness to Change -> Hort Engagement Since COVID	0.043	0.742	0.458
H2c: Self-Enhancement -> Hort Engagement Since COVID	**0.276**	4.216	0.000
H2d: Conservation -> Hort Engagement Since COVID	**0.203**	3.355	0.001
H3: Attitudes Towards Hort Growers -> Hort Engagement Pre-COVID	**0.422**	7.711	0.000
H4: Attitudes Towards Hort Growers -> Hort Engagement Since COVID	**0.221**	3.862	0.000
H5: Attitudes Towards COVID -> Hort Engagement Since COVID	**0.220**	3.604	0.000
H6: Hort Engagement Pre-COVID -> Grow/Process vs. Buy Hort Products Pre-COVID	**0.753**	24.442	0.000
H7: Hort Engagement Pre-COVID -> Grow/Process vs. Buy Hort Products Since COVID	**0.284**	3.289	0.001
H8: Hort Engagement Since COVID -> Grow/Process vs. Buy Hort Products Since COVID	**0.512**	5.986	0.000

Bold = *p* < 0.05.

## 4. Discussion

Factors determining the preferences of US consumers towards the growing and processing of horticultural products such as fruit, vegetables, wine and beer over buying them pre-COVID-19 as well as since the start of the coronavirus pandemic were examined in this study. Adequate fit was observed in the conceptual model, which had moderate explanatory power and strong predictive relevance. Engagement with horticulture prior to and after the occurrence of COVID-19 both represented key factors that influenced preferences for the growing and processing of fruit, vegetables, wine and beer over buying them in both the pre-COVID-19 and post-COVID-19 contexts. Engagement with horticulture, both before and after the occurrence of COVID-19 was influenced by all the proposed predictors apart from the value categorized as ‘Openness to change’. In other words, attitudes towards US growers and three of the values impacted engagement with horticulture before and after the occurrence of COVID-19 and attitudes towards COVID influenced post-COVID-19 horticultural engagement.

The human values results partially confirm findings from the extant literature within this topic area. Self-transcendence and self-enhancement are important predictors of pro-social values and values relating to environmental behavior and have been shown to be related to the motivation to participate in community or home gardening [11,16,38,49]. The negative but significant relationship of self-transcendence as a predictor for engagement with horticulture may be explained as follows: self-transcendence refers to a state in which individuals are able to look beyond themselves and adopt a larger perspective that includes concern for others [40,41]; however, engagement with horticulture and gardening is often necessary for more self-centered reasons. People may participate in gardening, food processing and food sharing because they may not have been in an economically strong situation and this may have also been the case even before the occurrence of COVID-19 [76,77]. Food insecurity, food control and the need for inclusion or therapy are important reasons why people seek engagement with horticultural activities and these aspects are often more related to acts of resilience and self-concern [16,77]. Similar reasons may explain why the value ‘Openness to change’ is not significant and that the value ‘Conservation’ is significant. Typically, when individuals engage in horticultural activities, they are often not looking for excitement, novelty or a challenge in life but are rather seeking stability, healing, harmony and hopefully a means to an end [11,16,77,78,79].

From a consumer’s perspective, the tradition, contribution and impact of a horticultural grower’s work is of relevance. Horticultural growers influence, direct and undertake production processes and are therefore in charge of the respective impacts of these on both people and environment [31]. Positive and negative public discourse about the contribution and externalities is likely to shape attitudes and, therefore, the level of consumer engagement with horticulture [15]. The occurrence of COVID-19 was definitely a turning point in terms of these negative attitudes as it allowed the horticultural sector to thrive, which is reflected in not only the stronger consumer interest in horticulture but also in the increased sales of plants and other horticultural materials [11,16].

The results pertaining to consumer attitudes towards COVID-19 and engagement support what has been shown within the extant literature in this topic area. It has been reported that extended stay-at-home periods and economic hardship drove engagement with horticulture in both community and home gardening, as well as in food sharing and processing [11,16,55,80,81]. Reservations, negative attitudes and shying away from engagement with horticultural activities may be related to not only health mandates, but also because horticultural engagement is not a solitary activity but one that requires some form of physical activity and contact with other people. Ultimately, the extent of engagement and pre-exposure and experience with growing food and other horticultural activities may determine the decision whether to grow or to buy.

## 5. Managerial Implications

The results obtained in this study provide value for managers of community gardens, horticultural properties and specialized food stores. The trend of growing and processing horticultural products such as fruit, vegetables, wine, and beer over buying them is of relevance to not only market gardens and garden centers but also to home improvement stores and retail nurseries that sell plants, tools, accessories and other materials for production and processing directly to the consumer [16,55]. Businesses may wish to extend their current offerings in terms of knowledge to be appealing to hobbyists who are seeking increased engagement with horticulture. This may include food production and processing advice and variety knowledge, particularly around the topics of which varieties are suitable for fresh consumption and processing. Offering physical and online workshops, where plants, equipment and processes are demonstrated, may be an opportunity to attract new consumers and expand on pre-existing customer relationships [59]. A way to attract younger consumers who are digital natives could be through augmented reality, as this provides the interactive experience of a real-world environment, where the objects that reside in the real world are enhanced by computer-generated perceptual information, sometimes across multiple sensory modalities [82]. This may allow visits into professional horticultural production facilities such as wineries and beer brewing facilities as well as home and community gardens, which could provide further inspiration to consumers.

## 6. Limitations and Suggestions for Future Research

One limitation of this research is the approach to data collection, namely using a crowdsourcing platform like Amazon Mechanical Turk. Amazon Mechanical Turk has been criticized in the past within academic circles due to the quality of the data and ethical issues surrounding the payment of workers [60,61]. This platform has been widely used over the past decade, in particular by researchers in psychology, organizational behavior, economics and marketing [61]. Using Amazon Mechanical Turk for data collection has been recognized as being equal to other forms of convenience sampling when considering data quality [60,61]. However, when comparing with national representative samples, a few aspects of the present M-Turk sample need to be critically acknowledged. The sample overall is younger and better educated and there are differences in income and geographical distribution. Respectively, the authors would set more detailed targets for age, gender, income and regions to match the most recent census. In addition, the authors would include questions that allow to distinguish whether survey participants live in rural or urban areas. It would be expected that people in rural areas may have fewer space constraints and a stronger connection to land and horticultural production.

A further limitation of the study is that sustainability and health consciousness were not specifically included in the model as predictors. In particular, the disruption caused by the coronavirus pandemic has led towards health and well-being trends, and some consumers have increased fruit and vegetable consumption or are demanding super foods. The inclusion of sustainability may have also impacted the findings, as fair and sustainably produced food is still demanded by consumers due to increased consumer awareness of food production systems. In addition, it needs to be critically evaluated whether the results of the present study would remain valid once consumers start living in an endemic world alongside the virus. Due to the risk of virus mutation and the occurrence of more infectious variants, it can be expected that supply chain disruptions and changes in food prices may impact consumers in the long term. This would particularly apply to those in the low-income strata who rely on gardening to access fresh produce.

Another research direction could be the examination of how COVID-19 has impacted specific consumer attitudes towards horticulture and the buy/grow balance. When making anecdotal comparisons of the descriptive statistics of these attitudes both before and during the pandemic (Table 2c), some attitudes seem to have changed more than others and examining such changes could be a fruitful line of enquiry. Further study of the underlying motivations for such preferences could also yield some interesting insights. The conceptual model presented in this study shows that this research examines a pertinent issue: a growing consumer interest in horticultural activities and gardening as a result of the COVID-19 pandemic. Future research could address the findings of attitudes towards growers from both a practitioner and a consumer perspective. This investigation could focus on social license to operate [52] and could compare businesses that enjoy the approval of consumers and society at large as well as businesses that need to improve in this area. A mixed-method study may be a suitable approach for such an investigation, allowing for both in-depth information and generalizability.

Further studies could build on work by Behe et al., (2022), who analyzed the motivation of three generational cohorts (baby boomers, millennials and Generation Z) to purchase plants during the coronavirus pandemic [55]. An extension of this research could focus on the ‘Silent generation’ and ‘Generation Alpha’. Both generational cohorts are of particular interest as they are widely ignored by academic studies. Members of the ‘Silent generation’ are likely to have had a crisis experience in their life and therefore have a different motivation towards being engaged with horticulture. In a similar manner ‘Generation Alpha’ are future consumers and have had their interests and experiences shaped by their millennial parents, who hold pro-social and pro-environmental values.

In addition, the findings related to horticultural engagement could be deepened in future studies by framing the study in a do-it-yourself plant parenting and sharing context. These are trends that have grown in popularity during the coronavirus pandemic and are predicted to remain so for the long term [11,59].

## Figures and Tables

**Table 1:** Demographic Information (*n* = 383).

	Freq	%	% US Census
Age (StDev: 0.940)			
18–24	18	4.7	12
25–34	215	56.1	18
35–44	104	27.2	16
45–54	27	7.0	16
55–64	14	3.7	17
65+	5	1.3	21
Total	383	100	100
Education (StDev: 0.927)			
Did not finish high school	6	1.6	11
Finished high school	46	12.0	27
Attended university	40	10.4	20
Bachelor’s degree	223	58.2	29
Postgraduate degree	68	17.8	13
Total	383	100	100
Household Annual Income (StDev: 1.141)			
USD0 to USD24,999	80	20.9	18
USD25,000 to USD49,999	117	30.5	20
USD50,000 to USD74,999	119	31.1	18
USD75,000 to USD99,999	40	10.4	13
USD100,000 or higher	27	7.0	31
Total	383	100	100
Gender (StDev: 0.501)			
Male	196	51.2	49
Female	187	48.8	51
Total	383	100	100
Region			
Northeast	83	21.7	17
South	90	23.5	38
Midwest	133	34.8	21
West	77	20.1	24
Total	383	100	100

**Table 2:** (**a**) Schwartz Values: descriptive stats, scale loadings, reliabilities and convergent validity. (**b**) Attitudes to US growers and COVID-19: descriptive stats, loadings, reliabilities and convergent validity. (**c**) Hort-engagement and grow vs. buy preferences: loadings, reliabilities and convergent validity.

Scales and Items	Mean	Std Dev	Factor Loadings	Cronbach’s Alpha	Composite Reliability	Average Variance Extracted
(**a**)						
Schwartz Value: Self-Enhancement				0.629	0.835	0.718
Importance of POWER (social power, authority, wealth)	4.89	1.68	0.924			
Importance of ACHIEVEMENT (success, capability, ambition, influence on people and events)	5.42	1.35	0.763			
Schwartz Value: Openness to Change				0.659	0.785	0.560
Importance of HEDONISM (gratification of desires, enjoyment in life, self-indulgence)	5.21	1.46	0.835			
Importance of STIMULATION (daring, a varied and challenging life, an exciting life)	5.27	1.39	0.850			
Importance of SELF-DIRECTION (creativity, freedom, curiosity, independence, choosing one’s own goals)	5.56	1.32	0.512			
Schwartz Value: Self-Transcendence				0.502	0.762	0.630
Importance of UNIVERSALISM (broad-mindedness, beauty of nature and arts, social justice, a world at peace, equality, wisdom, unity with nature, environmental protection)	5.56	1.24	0.963			
Importance of BENEVOLENCE (helpfulness, honesty, forgiveness, loyalty, responsibility)	5.51	1.30	0.576			
Schwartz Value: Conservation				0.736	0.843	0.644
Importance of TRADITION (respect for tradition, humbleness, accepting one’s portion in life, devotion, modesty)	5.36	1.37	0.851			
Importance of CONFORMITY (obedience, honoring parents and elders, self-discipline, politeness)	5.10	1.55	0.869			
Importance of SECURITY (national security, family security, social order, cleanliness, reciprocation of favors)	5.66	1.12	0.672			
(**b**)						
Attitudes towards US Growers				0.839	0.876	0.503
US growers have a longstanding tradition and lots of experience in growing sustainable apples	5.34	1.34	0.63			
US apple growers contribute to the care and maintenance of the landscape	5.44	1.25	0.682			
US apple growers make active contributions to preserve biodiversity	5.24	1.38	0.764			
US apple growers treat land resources responsibly	5.47	1.22	0.708			
Social pressure on apple growers should be increased as they are the main agents of climate change	4.98	1.55	0.743			
Social pressure on apple growers should be increased as they are the main agents of eutrophication	5.17	1.47	0.685			
US apple growers are environmentally conscious	5.32	1.37	0.745			
Attitudes towards COVID-19				0.772	0.844	0.522
I feel COVID-19 has changed our culture towards more inequality	5.04	1.53	0.837			
I feel COVID-19 has changed how we use technology	5.63	1.20	0.588			
I feel COVID-19 has changed our societal structures towards distance	5.43	1.29	0.722			
I feel COVID-19 has changed societal processes towards unfairness	5.32	1.35	0.722			
I feel COVID-19 has changed our economy towards more instability	5.37	1.36	0.723			
(**c**)						
Hort Engagement Pre-COVID				0.954	0.961	0.731
Before COVID-19—Growing fruit and vegetables in my own garden	4.42	1.80	0.811			
Before COVID-19—Participating in food and seed swaps	4.40	1.93	0.884			
Before COVID-19—Reading magazines and books about plants	4.56	1.79	0.861			
Before COVID-19—Watching YouTube clips on plant propagation	4.63	1.86	0.854			
Before COVID-19—Keeping a high stock of food	4.63	1.63	0.766			
Before COVID-19—Making my own bread, jam or juice	4.54	1.84	0.850			
Before COVID-19—Brewing wine or beer	4.06	2.05	0.884			
Before COVID-19—Keeping bees	4.17	2.09	0.877			
Before COVID-19—Participating in food sharing or other sharing opportunities	4.37	2.00	0.897			
Hort Engagement Since COVID				0.938	0.948	0.671
Since COVID-19—Growing fruit and vegetables in my own garden	4.55	1.78	0.786			
Since COVID-19—Participating in food and seed swaps	4.40	1.94	0.870			
Since COVID-19—Reading magazines and books about plants	4.50	1.75	0.832			
Since COVID-19—Watching YouTube clips on plant propagation	4.63	1.76	0.817			
Since COVID-19—Keeping a high stock of food	4.79	1.56	0.653			
Since COVID-19—Making my own bread, jam or juice	4.60	1.83	0.799			
Since COVID-19—Brewing wine or beer	3.97	2.01	0.873			
Since COVID-19—Keeping bees	4.17	2.04	0.838			
Since COVID-19—Participating in food sharing or other sharing opportunities	4.21	1.95	0.882			
Grow vs. Buy Fruit Preference Pre-COVID				0.947	0.959	0.825
Before COVID-19, did you prefer buying or growing your own apples and other pip fruit?	51.78	29.99	0.914			
Before COVID-19, did you prefer buying or growing your own berries and other soft fruit?	50.07	29.69	0.937			
Before COVID-19, did you prefer buying or making your own wine or beer?	46.18	31.97	0.891			
Before COVID-19, did you prefer buying or growing your own lemons and other citrus fruit?	50.88	30.87	0.914			
Before COVID-19, did you prefer buying or growing your own vegetables?	59.59	29.19	0.885			
Grow vs. Buy Fruit Preference Since COVID				0.945	0.958	0.820
Since COVID-19, do you prefer buying or growing your own apples and other pip fruit?	52.64	30.32	0.927			
Since COVID-19, do you prefer buying or growing your own berries and other soft fruit?	51.68	29.85	0.928			
Since COVID-19, do you prefer buying or making your own wine or beer?	46.16	31.94	0.886			
Since COVID-19, do you prefer buying or growing your own lemons and other citrus fruits?	52.01	31.01	0.915			
Since COVID-19, do you prefer buying or growing your own vegetables?	59.78	28.77	0.871			

Note: (**a**) Adapted from Lindeman and Verkasalo (2005) [63]. (**b**) Items developed by the authors following Bir et al., (2021), Mullins et al., (2021) and Bulgari et al., (2021) and adapted from Klein (2011) [1,11,16]. (**c**) Items developed by the authors following Bulgari et al., (2021) [11].

**Table 3:** Scale Discriminant Validity.

Fornell–Larcker Criterion	A	B	C	D	E	F	G	H	I	J
(A) Attitudes towards COVID	0.723									
(B) Attitudes towards US growers	0.478	0.709								
(C) Conservation	0.483	0.551	0.804							
(D) Grow/Make vs. Buy Hort Pre-COVID	0.444	0.409	0.389	0.908						
(E) Grow/Make vs. Buy Hort Since COVID	0.428	0.398	0.410	0.947	0.906					
(F) Hort Engagement Pre-COVID	0.439	0.597	0.462	0.753	0.730	0.855				
(G) Hort Engagement Since COVID	0.534	0.561	0.552	0.767	0.759	0.870	0.819			
(H) Openness to Change	0.485	0.533	0.516	0.420	0.426	0.415	0.474	0.756		
(I) Self Enhancement	0.527	0.553	0.604	0.479	0.480	0.547	0.619	0.624	0.849	
(J) Self-Transcendence	0.413	0.384	0.499	0.128	0.159	0.152	0.251	0.510	0.325	0.810
**Heterotrait–Monotrait Ratio**										
Attitudes towards US growers	0.599									
Conservation	0.629	0.675								
Grow/Buy Hort Pre-COVID	0.478	0.422	0.439							
Grow/Buy Hort Since COVID	0.462	0.408	0.465	1.000						
Hort Engagement Pre-COVID	0.484	0.633	0.520	0.789	0.765					
Hort Engagement Since COVID	0.604	0.603	0.640	0.809	0.801	0.915				
Openness to Change	0.731	0.699	0.747	0.448	0.464	0.463	0.551			
Self-Enhancement	0.745	0.752	0.875	0.587	0.588	0.672	0.774	0.948		
Self-Transcendence	0.739	0.614	0.865	0.184	0.229	0.254	0.382	1.025	0.634	

## Data Availability

The data presented in this study are available on request from the corresponding author.
